# Area-level socioeconomic status is positively correlated with glioblastoma incidence and prognosis in the United States

**DOI:** 10.3389/fonc.2023.1110473

**Published:** 2023-03-17

**Authors:** Maria P. Gorenflo, Alan Shen, Erin S. Murphy, Jennifer Cullen, Jennifer S. Yu

**Affiliations:** ^1^Cleveland Clinic Lerner College of Medicine, Case Western Reserve University, Cleveland, OH, United States; ^2^Department of Radiation Oncology, Cleveland Clinic Foundation, Cleveland, OH, United States; ^3^Department of Population and Quantitative Health Sciences, Case Comprehensive Cancer Center, Case Western Reserve University, Cleveland, OH, United States; ^4^Department of Cancer Biology, Lerner Research Institute, Cleveland Clinic Foundation, Cleveland, OH, United States

**Keywords:** glioblastoma, socioeconomic status, social determinants, public health, health disparities, race, ethnicity, survival

## Abstract

In the United States, an individual’s access to resources, insurance status, and wealth are critical social determinants that affect both the risk and outcomes of many diseases. One disease for which the correlation with socioeconomic status (SES) is less well-characterized is glioblastoma (GBM), a devastating brain malignancy. The aim of this study was to review the current literature characterizing the relationship between area-level SES and both GBM incidence and prognosis in the United States. A query of multiple databases was performed to identify the existing data on SES and GBM incidence or prognosis. Papers were filtered by relevant terms and topics. A narrative review was then constructed to summarize the current body of knowledge on this topic. We obtained a total of three papers that analyze SES and GBM incidence, which all report a positive correlation between area-level SES and GBM incidence. In addition, we found 14 papers that focus on SES and GBM prognosis, either overall survival or GBM-specific survival. Those studies that analyze data from greater than 1,530 patients report a positive correlation between area-level SES and individual prognosis, while those with smaller study populations report no significant relationship. Our report underlines the strong association between SES and GBM incidence and highlights the need for large study populations to assess SES and GBM prognosis to ideally guide interventions that improve outcomes. Further studies are needed to determine underlying socio-economic stresses on GBM risk and outcomes to identify opportunities for intervention.

## 1 Introduction

Glioblastoma (GBM) is a grade IV glioma that is the most common malignant primary brain tumor, comprising 60% of all cases with an annual incidence of 5.0 per 100,000 (1). According to the Central Brain Tumor Registry of the United States (CBTRUS), over 63,000 Americans were diagnosed with GBM between 2015 and 2019 (2). Standard of care currently involves surgical resection followed by radiation therapy and chemotherapy, but even with this aggressive treatment, the overall five-year survival rate is only 5% (3). Many efforts have been made over the years to better elucidate the underlying biological and physiological basis of GBM, but the socioeconomic factors underlying disease development and outcomes have received less attention. When considering the United States, where access to quality healthcare largely depends on an individual’s economic resources, such as income and health insurance, socioeconomic factors are particularly crucial to analyze with respect to the incidence and prognosis of any disease, including GBM. Previous studies have uncovered correlations between area-level socioeconomic metrics and either GBM or other gliomas (4–7) in the United States on the regional, state, and national levels. Here, we report a comprehensive review to synthesize current knowledge regarding the correlations between socioeconomic status (SES) and GBM incidence and prognosis in the United States.

## 2 Methods

**Figure 1:**
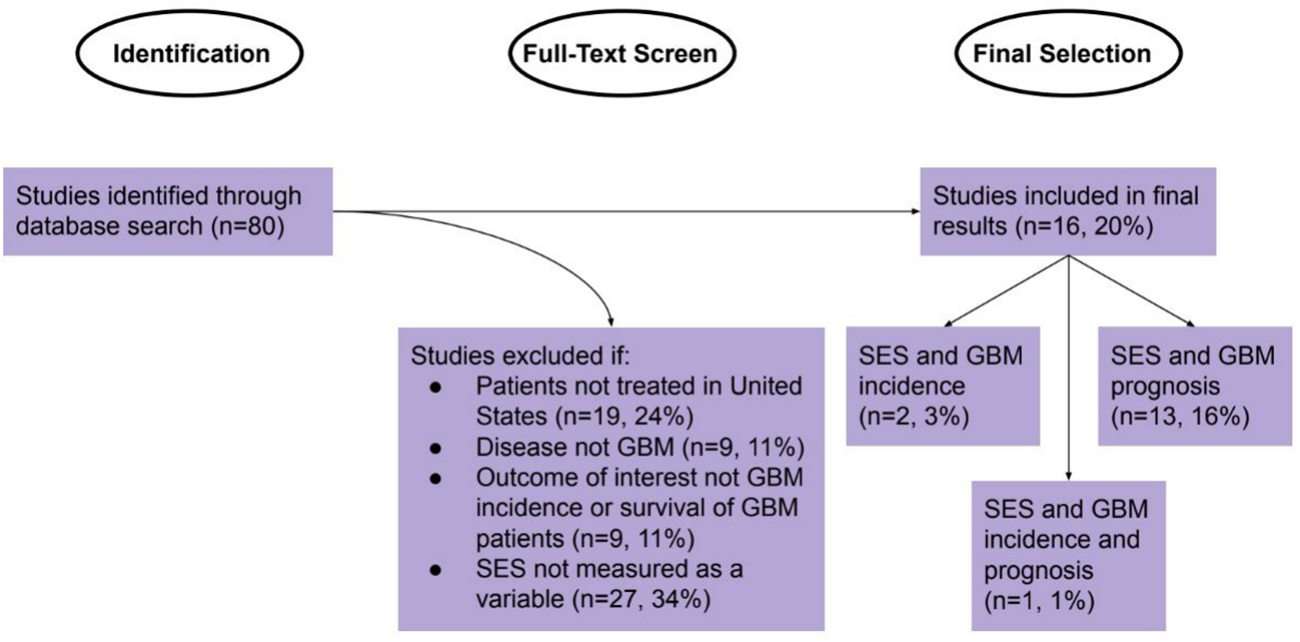
Identification, Screening, and Selection of Studies for Review.

A review of studies was conducted by querying those published between January 1, 1990, and June 1, 2022, on Google Scholar (Google Scholar, RRID : SCR_008878), PubMed (PubMed, RRID : SCR_004846), Scopus (Scopus, RRID : SCR_022559), and Web of Science (Web of Science, RRID : SCR_022706). The Cochrane database (Cochrane Library, RRID : SCR_013000) was searched to determine that no identical review had previously been conducted. The terms used for these searches were “socioeconomic status,” “SES,” “income,” “glioblastoma,” and “GBM.” All studies found using this method were initially recorded, then further filtered. The criteria for selection were studies that analyzed patients in the United States and characterized a relationship between SES and either GBM incidence, GBM-specific survival, or overall survival (OS) in patients with GBM. SES involved either a measurement of income itself, an aggregate index for SES involving income, defined economic variables (such as education, poverty, unemployment, rent, and house value measures), or a self-identified SES measurement whose components were not specified. SES was also measured on either the individual or area level. Use of these search criteria led to identification of 80 studies. These studies were then individually reviewed by full-text analysis and synthesized in a narrative review. Studies were excluded if the target population was outside of the United States, if the cancer analyzed was not GBM, if SES was not included as a variable, or if the outcome measured was not GBM incidence or disease-specific or overall survival of GBM patients (**Figure 1**). The full-text analysis yielded a final list of 16 studies (three for GBM incidence and 14 for GBM prognosis, including one study that measured both) that were included in the Results. The following characteristics were recorded: title, year, authors, participants, data sources, SES measure, methodology, dependent outcomes, and key findings.

## 3 Results

Overall, 16 studies met the criteria for inclusion: two for SES and GBM incidence, 13 for SES and GBM specific survival or OS, and one for SES and both GBM incidence and OS. The GBM incidence papers analyzed between 3,832 and 45,696 patients and were published between 2005 and 2019 (**Table 1**). The GBM survival and OS papers analyzed between 116 and 61,346 patients and were published between 2007 and 2022; 12 papers measured OS as their outcome, one measured GBM-specific mortality, and one measured both (**Table 2**).

**Table 1:** Area-level socioeconomic status and GBM incidence.

Title	Year	Authors	Participants (# and Population)	Data Sources	SES Measure	Methods	Outcomes	Findings
A population-based description of glioblastoma multiforme in Los Angeles County, 1974–1999	2005	Chakrabarti et al. (8)	3,832 patients in Los Angeles County diagnosed with GBM between 1974 and 1999	Los Angeles County Cancer Surveillance Program	Census tract median household income and educational attainment combined to create SES assignment	Retrospective analysis, multivariate analysis (Poisson regression)	GBM incidence	Those in the highest SES tertile had a significantly greater chance of developing GBM than those in the middle and lowest tertiles.
Socioeconomic status and glioblastoma risk: a population-based analysis	2015	Porter et al. (9)	26,481 GBM patients diagnosed 2000-2010 without prior low-grade glioma	SEER	SES quintile (based on median household income, proportion below 150% poverty line, proportion unemployed >16, proportion w/blue-collar jobs, median rent, median housing value, educational index) by census tract	Retrospective analysis Poisson regression modeling	GBM incidence	Highest SES quintile has a GBM incidence ratio of 1.45 compared to the lowest SES quintile.
Glioma incidence and survival variation by county-level socioeconomic measures	2019	Cote et al. (10)	45,696 GBM patients in the CBTRUS database 2011-2015, 90% histologically confirmed	CBTRUS	County-level SES incorporating education, employment, poverty, median income, occupation	Retrospective analysis, average age-adjusted incidence rates, incidence rates ratios to compare differences	GBM incidence	GBM incidence greatest in highest SES quintile counties.

**Table 2:** Area-level socioeconomic status and survival in GBM patients.

Title	Year	Authors	Participants (# and Population)	Data Sources	SES Measure	Methods	Outcomes	Findings
Racial/ethnic differences in survival among elderly patients with a primary glioblastoma	2007	Barnholtz- Sloan et al. (11)	1,530 patients diagnosed with GBM >=66 years from 6/1/91 to 12/31/99 on Medicare	SEER	Median household income by 1990 census tract (low if under $30k, high if over $30k)	Retrospective Kaplan-Meier and multivariable Cox analyses	Overall survival	No significant difference in survival by high vs. low area median income.
Socioeconomic status predicts survival in patients with newly diagnosed glioblastoma	2014	Leeper & Johnson (12)	26,841 GBM patients diagnosed 2000-2010 without prior low-grade glioma	SEER	Census-tract SES quintile based on Yost index: occupation, unemployment, poverty, income, education, and house values	Retrospective Kaplan-Meier analysis, univariable and multivariable Cox proportional hazards regression model	Median overall survival	Significantly higher GBM survival in highest quintile compared to middle and lowest quintiles Strong association between census tract SES and survival in multivariable model with age, sex, race/ethnicity, radiation, and surgery type.
Socioeconomic status does not affect prognosis in patients with glioblastoma multiforme	2016	Kasl et al. (13)	218 patients with histologically confirmed GBM treated at Vanderbilt University Medical Center 2000-2014, excluding children and incarcerated patients	Vanderbilt University Medical Center EHR	Zip code tabulation area average income split into tertiles: <250%, 250-500%, and >500% national poverty level	Retrospective analysis, multivariate Cox proportional hazards analysis	Median survival time	No relationship between SES and survival.
Disparities in receipt of modern concurrent chemoradiotherapy in glioblastoma	2016	Rhome et al. (14)	28,279 GBM patients diagnosed 1998-2012 that underwent surgical resection or biopsy; excluded if multifocal disease	NCDB	Median household income by zip code quartile	Retrospective univariable and multivariable Cox analysis	Overall survival	>$46k area income has greater overall survival than <$30k area income in uni- and multivariable analyses.
Socioeconomic status and survival in glioblastoma	2016	Trikalinos et al. (15)	131 patients treated for GBM at University of Maryland 2001-2012	University of Maryland electronic health records	Area-level SES from census tract poverty percentage as done by NCI SEER	Retrospective survival analysis	Overall survival	No association between overall survival and SES.
Hispanic ethnicity and socioeconomic status are independently associated with improved prognosis in glioblastoma patients	2017	Moore et al. (16)	16,180 Florida adults diagnosed with GBM 1981-2013	Florida Cancer Data System	SES but not specified (perhaps US Census)	Retrospective multivariate Cox regression model	Overall survival	Higher SES associated with increased survival.
Patterns and disparities of care in glioblastoma	2019	Dressler et al. (17)	61,346 GBM patients diagnosed 1998-2011 age 20+, diagnosis by pathology, imaging, or direct visualization	NCDB	Zip code median household income	Retrospective multivariate Cox proportional hazards	All-cause mortality	Mortality decreased if area income > $46,000 compared to < $46,000.
Tumor-induced mortality in adult primary supratentorial glioblastoma multiforme with different age subgroups	2019	Shu et al. (18)	20,550 adult primary supratentorial GBM patients diagnosed 2000-2013; patients with previous malignancy excluded	SEER	SES tertile (source not specified)	Retrospective competing risk regression, Cox regression	Risk of GBM-induced mortality	The risk of GBM mortality is significantly lower in the highest SES tertile compared to the lower two.
Glioma incidence and survival variation by county-level socioeconomic measures	2019	Cote et al. (10)	16,059 adult GBM patients diagnosed 2000-2015 with histologic confirmation	SEER	County-level SES quintile, incorporating education, employment, poverty, median income, occupation	Retrospective Cox proportional hazards model	Median overall survival	GBM survival greater in highest SES quintile compared to lowest (adjusted for age, extent of resection, receipt of chemoradiation treatment).
Community economic factors influence outcomes for patients with primary malignant glioma	2020	Bower et al. (19)	312 patients with a primary malignant glioma (223 grade IV) histologically confirmed 18+ years of age who received primary treatment 1999-2007 at Wake Forest Baptist	Wake Forest Baptist Comprehensive Cancer Registry	Zip code median income - if above state median then high income, if below then low income	Retrospective 2-sample t-test Kaplan-Meier survival probability	Overall survival	No significant difference in overall survival between high and low income cohorts for grade IV glioma.
Real-world evaluation of the impact of radiotherapy and chemotherapy in elderly patients with glioblastoma based on age and performance status	2020	Al Feghali et al. (20)	48,540 histologically confirmed GBM patients 2004-2015 ages 60+	NCDB	Median income quartile (not specified)	Retrospective multivariate analysis	Overall survival	Worse overall survival associated with lower income.
Identifying disparities in care in treating glioblastoma: a retrospective cohort study of patients treated at a safety-net versus private hospital setting	2020	Wang et al. (21)	116 patients from two USC hospitals treated between 2010 and 2014 without previous glioma treatment	Records from USC Norris and LA County USC Medical Centers	Area household income from 2016 American Community Survey (low income<$50k, high income >$50k) (by census tract)	Retrospective univariable and multivariable Cox proportional hazards analysis	Overall survival	Income not associated with overall survival in univariable or multivariable analysis.
Racial and socioeconomic disparities differentially affect overall and cause-specific survival in glioblastoma	2020	Liu et al. (22)	28,952 adult patients diagnosed with histologically confirmed GBM 2005-2016	SEER	County median income	Retrospective Kaplan-Meier survival analysis	Overall survival, GBM mortality, non-GBM mortality	Overall survival increased with increase in county income; largely due to differences in non-GBM mortality, as GBM mortality not significantly different between different income brackets.
Predicting access to postoperative treatment after glioblastoma resection: an analysis of neighborhood-level disadvantage using the Area Deprivation Index (ADI)	2022	Rivera Perla et al. (23)	434 GBM patients who underwent index resection 2012-2017 ages 18+ at Rhode Island Hospital or Mayo Clinic	Rhode Island Hospital and Mayo Clinic databases	Area of Deprivation Index percentile based on address; split into high area-deprivation index (top 66%, upper half) and low (bottom 33%, lower half)	Retrospective multivariable regression model	Overall survival	No difference in overall survival between high and low area-deprivation index.

All papers utilized area-level, not individual-level, SES measures; three examined the county level, four at the zip code level, six at the census tract level, one at the address level using a pre-existing index, and three were not specified. SES was measured solely by income in eight of the papers – either average, median, or quintile/quartile/tertile. Six papers utilized an aggregate SES metric involving income as well as other variables, and two papers did not specify their SES measurement. All three papers that correlated SES to incidence of GBM found a greater incidence of GBM in higher SES regions (**Table 1**). Of the 14 papers that correlated SES to patient survival, the eight with greater than 1,530 patients analyzed found that patients living in regions with higher SES experienced longer survival on average. The six studies with ≤ 1,530 patients found no significant relationship between area-level SES and survival in GBM patients, highlighting the need for larger patient studies (**Table 2**).

With respect to patient population, nine papers analyzed SES and either GBM incidence or prognosis from national databases. Specifically, six papers obtained GBM patient information from the Surveillance, Epidemiology, and End Results Program (SEER), three papers acquired data from the National Cancer Database (NCDB), and one paper included incidence data from the CBTRUS. Another five papers obtained data from university hospital records, and the two remaining papers analyzed GBM records from state- or city-level databases. GBM incidence studies, in aggregate, contained data for patients diagnosed between 1974 and 2015, while GBM prognosis studies included patient data between 1981 and 2017.

## 4 Discussion

This review uncovered a positive correlation between both area-level SES and GBM incidence, as well as area-level SES and survival in GBM patients in studies with larger sample sizes. When considering these results, it is important to keep in mind that SES is measured at the level of a geographic region, so it cannot be applied to individual-level SES associations with GBM incidence or prognosis. Individuals who live in an area with a higher SES experience a higher rate of GBM incidence, but an individual with a higher SES does not necessarily display a greater risk of developing GBM.

When considering the positive association between SES and GBM incidence, it is important to examine the interface between these variables and age distribution. Higher SES areas tend to have a greater life expectancy (24), and the average age of onset for GBM is 64 years of age (25). However, all three papers that examined this relationship utilized age-adjusted GBM incidence (8–10), so age differences between high and low SES regions should not explain this relationship. Another important consideration is race and ethnicity, as there is ample evidence that non-Hispanic whites are the most at-risk for developing GBM (2, 10), and that this group is over-represented in high SES regions. However, two of the papers analyzing SES and GBM incidence controlled for race in their incidence calculations (8, 10), and the third stratified incidence calculations by race, demonstrating that non-Hispanic white, white Hispanic, and black patients experience greater GBM incidence as area-level SES increases (9). Additionally, one may consider that patients in high SES regions may have greater access to diagnostic modalities that reveal GBM, artificially increasing GBM diagnoses in these regions. Clinical suspicion for GBM begins with apparent space-occupying lesions on computer tomography or magnetic resonance imaging, and GBM typically progresses to severe signs and symptoms, such as seizure, even if initial presentation was non-specific (26). Therefore, it is unlikely that many patients would progress through their entire disease course without proper diagnosis either pre- or post-mortem. Overall, it remains unclear why there is a direct relationship between SES and GBM incidence.

The positive association between SES and survival in GBM patients, on the other hand, was only observed in studies with larger sample sizes. This is likely due to the variability in metrics used to assess SES and the heterogeneity in SES at different geographical (e.g., census tract, zip code, versus county) regions. This association could be explained by a multitude of factors, the first being type of health care insurance. Insured GBM patients have a better prognosis than those uninsured (27), patients with private insurance survive longer than those on Medicaid (28), and non-Medicaid patients survive longer than those on Medicaid or who are uninsured (29). Therefore, perhaps individuals in high SES areas are more likely to have private insurance, leading to better access to care and therefore better prognosis.

Another factor explaining this relationship may be the type of treatment received. GBM patients living in higher SES regions have a greater chance of receiving radiation, and those who receive this treatment have greater OS (30–32). The same can be said for GBM patients who receive triple therapy (surgery, radiation, and chemotherapy) (33, 34). An additional consideration is clinical trial participation: those living in greater SES regions are more likely to participate in clinical trials (23), which is correlated with improved OS (35) and allows for more salvage therapy options. Interestingly, it does not appear that Karnofsky Performance Status differs between those living in high and low SES regions (19, 23). Therefore, this relationship cannot be explained by patients in low SES regions presenting with more severe disease than those in high SES regions and may instead reflect differences in access to quality care.

There are several limitations to the conclusions that can be drawn from this review. First, due to the ecological fallacy, interpretation must remain on the area level and cannot be applied to individuals with high or low SES. Second, this study is not a systematic review or meta-analysis, as it was not anticipated that there would be many studies that would qualify for quantitative analysis. Consequently, it is possible that additional qualifying studies exist that were missed by the identification process. Third, while there was no overlap in patient data in **Table 1** (SES and GBM incidence), there is likely substantial patient overlap in **Table 2** (SES and GBM prognosis), as multiple studies included either SEER or NCDB data in varying time periods that overlapped between 2005 and 2010. Therefore, there are likely redundant conclusions pulled from approximately half of these studies (including 10, 12, 14, 17, 18, 20, 22). It is critical to note that while there appears to be a positive correlation between area-level SES and GBM prognosis, several of the studies characterizing this relationship analyze many of the same patients. Finally, due to publication bias, it is possible that more studies than those included here were conducted that found no association between area-level SES and GBM incidence or survival but remain unpublished due to negative results.

Placing these results in a broader context, the relationship between area-level SES and cancer incidence appears to depend on the cancer site in question. Past studies reveal that the risk of gastric, colorectal, larynx, cervix, penile, and liver cancer are greater in low SES regions, whereas the risk of melanoma, thyroid, and testicular cancer are greater in high SES regions (36–39). On the other hand, it appears that there is a positive correlation between cancer prognosis and area-level SES across cancer type, including breast cancer (40), Hodgkin lymphoma (41), non-small cell lung cancer (42), liver, kidney, colorectal, and prostate cancers (43), and various childhood cancers (44). Therefore, the relationship between SES and GBM prognosis is comparable to other cancer sites.

In this review, both greater GBM incidence and survival of GBM patients in high SES regions were observed, specifically in studies with larger sample sizes. As research continues to be conducted on the social determinants of health related to GBM, it will be important to utilize these findings to improve patient outreach, clinical trial enrollment, and education in those areas where patients are more likely to develop GBM or exhibit worse prognosis from the disease. Hopefully, as treatments for GBM become more effective, such interventions will reduce disparities in health care and outcomes in those living in regions of varying SES.

## Author contributions

JY conceived of and provided critical edits for the manuscript. MG performed literature searches and drafted the manuscript. AS performed literature searches and provided edits for the manuscript. EM and JC provided major edits for the manuscript. All authors contributed to the article and approved the submitted version.

## References

[1] Ostrom QT Cote DJ Ascha M Kruchko C Barnholtz-Sloan JS. Adult glioma incidence and survival by race or ethnicity in the united states from 2000 to 2014. JAMA Oncol (2018) 4(9):1254–62. 10.1001/jamaoncol.2018.1789 PMC6143018 29931168

[2] Ostrom QT Price M Neff C Cioffi G Waite KA Kruchko C. CBTRUS statistical report: Primary brain and other central nervous system tumors diagnosed in the united states in 2015–2019. Neuro-Oncol (2022) 24(Supplement_5):v1–95. 36196752 10.1093/neuonc/noac202 PMC9533228

[3] Alexander BM Cloughesy TF. Adult glioblastoma. J Clin Oncol (2017) 35(21):2402–9. 10.1200/JCO.2017.73.0119 28640706

[4] Ryan CS Juhn YJ Kaur H Wi CI Ryu E King KS. Long-term incidence of glioma in Olmsted county, Minnesota, and disparities in postglioma survival rate: A population-based study. Neuro-Oncol Pract (2020) 7(3):288–98. 10.1093/nop/npz065 PMC7274190 32537178

[5] Deb S Pendharkar AV Schoen MK Altekruse S Ratliff J Desai A. The effect of socioeconomic status on gross total resection, radiation therapy and overall survival in patients with gliomas. J Neurooncol (2017) 132(3):447–53. 10.1007/s11060-017-2391-2 28258423

[6] Demers PA Vaughan TL Schommer RR. Occupation, socioeconomic status, and brain tumor mortality: A death certificate-based case-control study. J Occup Med (1991) 33(9):1001–6. 1660541

[7] Plascak JJ Fisher JL. Area-based socioeconomic position and adult glioma: A hierarchical analysis of surveillance epidemiology and end results data. PloS One (2013) 8(4):e60910. 23585860 10.1371/journal.pone.0060910 PMC3622005

[8] Chakrabarti I Cockburn M Cozen W Wang YP Preston-Martin S. A population-based description of glioblastoma multiforme in Los Angeles county, 1974–1999. Cancer (2005) 104(12):2798–806. 10.1002/cncr.21539 16288487

[9] Porter AB Lachance DH Johnson DR. Socioeconomic status and glioblastoma risk: A population-based analysis. Cancer Causes Control (2015) 26(2):179–85. 10.1007/s10552-014-0496-x 25421378

[10] Cote DJ Ostrom QT Gittleman H Duncan KR CreveCoeur TS Kruchko C. Glioma incidence and survival variations by county-level socioeconomic measures. Cancer (2019) 125(19):3390–400. 10.1002/cncr.32328 PMC6744292 31206646

[11] Barnholtz-Sloan JS Maldonado JL Williams VL Curry WT Rodkey EA Barker FG. Racial/ethnic differences in survival among elderly patients with a primary glioblastoma. J Neurooncol (2007) 85(2):171. 17530174 10.1007/s11060-007-9405-4

[12] Leeper H Johnson D. P17.43: SOCIOECONOMIC STATUS PREDICTS SURVIVAL IN PATIENTS WITH NEWLY DIAGNOSED GLIOBLASTOMA. Neuro-Oncol (2014) 16(Suppl 2):ii97–7.

[13] Kasl RA Brinson PR Chambless LB. Socioeconomic status does not affect prognosis in patients with glioblastoma multiforme. Surg Neurol Int (2016) 7(Suppl 11):S282–90. 10.4103/2152-7806.181985 PMC4866060 27217966

[14] Rhome R Fisher R Hormigo A Parikh RR. Disparities in receipt of modern concurrent chemoradiotherapy in glioblastoma. J Neurooncol (2016) 128(2):241–50. 10.1007/s11060-016-2101-5 26970981

[15] Trikalinos NA Kwok Y Goloubeva O Mehta M Sausville E. Socioeconomic status and survival in glioblastoma. Int J Clin Exp Med (2016) 9(2):4131–6.

[16] Moore KJ Richardson A Koru-Sengul T Ivan ME. 164 Hispanic ethnicity and socioeconomic status are independently associated with improved prognosis in glioblastoma patients. Neurosurgery (2017) 64(CN_suppl_1):241.

[17] Dressler EV Liu M Garcia CR Dolecek TA Pittman T Huang B. Patterns and disparities of care in glioblastoma. Neuro-Oncol Pract (2019) 6(1):37–46. 10.1093/nop/npy014 PMC6352755 30740232

[18] Shu C Yan X Zhang X Wang Q Cao S Wang J. Tumor-induced mortality in adult primary supratentorial glioblastoma multiforme with different age subgroups. Future Oncol (2019) 15(10):1105–14. 10.2217/fon-2018-0719 30880453

[19] Bower A Hsu FC Weaver KE Yelton C Merrill R Wicks R. Community economic factors influence outcomes for patients with primary malignant glioma. Neuro-Oncol Pract (2020) 7(4):453–60. 10.1093/nop/npaa010 PMC7393281 32765895

[20] Al Feghali KA Buszek SM Elhalawani H Chevli N Allen PK Chung C. Real-world evaluation of the impact of radiotherapy and chemotherapy in elderly patients with glioblastoma based on age and performance status. Neuro-Oncol Pract (2020) 8(2):199–208. 10.1093/nop/npaa064 PMC8049423 33898053

[21] Wang T Pham A Yoo S Attenello FJ Jennelle R Wagle N. Identifying disparities in care in treating glioblastoma: A retrospective cohort study of patients treated at a safety-net versus private hospital setting. World Neurosurg (2020) 137:e213–20. 10.1016/j.wneu.2020.01.133 PMC8908807 32001415

[22] Liu EK Yu S Sulman EP Kurz SC. Racial and socioeconomic disparities differentially affect overall and cause-specific survival in glioblastoma. J Neurooncol (2020) 149(1):55–64. 32617722 10.1007/s11060-020-03572-y

[23] Rivera Perla KM Tang OY Durfey SNM Vivas-Buitrago T Sherman WJ Parney I. Predicting access to postoperative treatment after glioblastoma resection: an analysis of neighborhood-level disadvantage using the area deprivation index (ADI). J Neurooncol (2022) 158(3):349–357. doi: 10.1007/s11060-022-04020-9 35503190

[24] Chetty R Stepner M Abraham S Lin S Scuderi B Turner N. The association between income and life expectancy in the united states, 2001-2014. JAMA (2016) 315(16):1750–66. 10.1001/jama.2016.4226 PMC4866586 27063997

[25] Ladomersky E Scholtens DM Kocherginsky M Hibler EA Bartom ET Otto-Meyer S. The coincidence between increasing age, immunosuppression, and the incidence of patients with glioblastoma. Front Pharmacol (2019) 10:200. doi: 10.3389/fphar.2019.00200 30971917 PMC6446059

[26] McKinnon C Nandhabalan M Murray SA Plaha P. Glioblastoma: Clinical presentation, diagnosis, and management. BMJ (2021) 374:n1560. 34261630 10.1136/bmj.n1560

[27] Chandra A Rick JW Dalle Ore C Lau D Nguyen AT Carrera D. Disparities in health care determine prognosis in newly diagnosed glioblastoma. Neurosurg Focus FOC (2018) 44(6):E16. 10.3171/2018.3.FOCUS1852 PMC6105315 29852776

[28] Brown DA Himes BT Kerezoudis P Chilinda-Salter YM Grewal SS Spear JA. Insurance correlates with improved access to care and outcome among glioblastoma patients. Neuro-Oncol (2018) 20(10):1374–82. 10.1093/neuonc/noy102 PMC6120360 29893906

[29] Rong X Yang W Garzon-Muvdi T Caplan JM Hui X Lim M. Influence of insurance status on survival of adults with glioblastoma multiforme: A population-based study. Cancer (2016) 122(20):3157–65. 10.1002/cncr.30160 27500668

[30] Pollom EL Fujimoto DK Han SS Harris JP Tharin SA Soltys SG. Newly diagnosed glioblastoma: Adverse socioeconomic factors correlate with delay in radiotherapy initiation and worse overall survival. J Radiat Res (Tokyo) (2018) 59(suppl_1):i11–8. 10.1093/jrr/rrx103 PMC5868191 29432548

[31] Aizer AA Ancukiewicz M Nguyen PL Shih HA Loeffler JS Oh KS. Underutilization of radiation therapy in patients with glioblastoma. Cancer (2014) 120(2):238–43. 10.1002/cncr.28398 24122361

[32] Huang B Dolecek TA Chen Q Garcia CR Pittman T Villano JL. Characteristics and survival outcomes associated with the lack of radiation in the treatment of glioblastoma. Med Oncol (2018) 35(5):74. 29667068 10.1007/s12032-018-1134-3

[33] Lu VM Shah AH Eichberg DG Quinones-Hinojosa A Esquenazi Y Komotar RJ. Geographic disparities in access to glioblastoma treatment based on Hispanic ethnicity in the united states: Insights from a national database. J Neurooncol (2020) 147(3):711–20. 10.1007/s11060-020-03480-1 32236779

[34] Lu VM Lewis CT Esquenazi Y. Geographic and socioeconomic considerations for glioblastoma treatment in the elderly at a national level: A US perspective. Neuro-Oncol Pract (2020) 7(5):522–30. 10.1093/nop/npaa029 PMC7516121 33014393

[35] Field KM Rosenthal MA Yilmaz M Tacey M Drummond K. Comparison between poor and long-term survivors with glioblastoma: Review of an Australian dataset. Asia Pac J Clin Oncol (2014) 10(2):153–61. 10.1111/ajco.12076 23701649

[36] Boscoe FP Johnson CJ Sherman RL Stinchcomb DG Lin G Henry KA. The relationship between area poverty rate and site-specific cancer incidence in the united states. Cancer (2014) 120(14):2191–8. 10.1002/cncr.28632 PMC4232004 24866103

[37] Doubeni CA Doubeni AR Myers AE. Diagnosis and management of ovarian cancer. Am Fam Physician (2016) 93(11):937–44. 27281838

[38] Uthman OA Jadidi E Moradi T. Socioeconomic position and incidence of gastric cancer: A systematic review and meta-analysis. J Epidemiol Community Health (2013) 67(10):854. 23929615 10.1136/jech-2012-201108

[39] Shebl FM Capo-Ramos DE Graubard BI McGlynn KA Altekruse SF. Socioeconomic status and hepatocellular carcinoma in the united states. Cancer Epidemiol Biomarkers Prev (2012) 21(8):1330–5. 10.1158/1055-9965.EPI-12-0124 PMC3647693 22669949

[40] Smith EC Ziogas A Anton-Culver H. Delay in surgical treatment and survival after breast cancer diagnosis in young women by Race/Ethnicity. JAMA Surg (2013) 148(6):516–23. 10.1001/jamasurg.2013.1680 23615681

[41] Smith EC Ziogas A Anton-Culver H. Association between insurance and socioeconomic status and risk of advanced stage Hodgkin lymphoma in adolescents and young adults. Cancer (2012) 118(24):6179–87. 10.1002/cncr.27684 22736071

[42] Tannenbaum SL Koru-Sengul T Zhao W Miao F Byrne MM. Survival disparities in non–small cell lung cancer by race, ethnicity, and socioeconomic status. Cancer J (2014) 20(4):237–245. 25098282 10.1097/PPO.0000000000000058

[43] Kish JK Yu M Percy-Laurry A Altekruse SF. Racial and ethnic disparities in cancer survival by neighborhood socioeconomic status in surveillance, epidemiology, and end results (SEER) registries. JNCI Monogr (2014) 2014(49):236–43. 10.1093/jncimonographs/lgu020 PMC4841168 25417237

[44] Gupta S Wilejto M Pole JD Guttmann A Sung L. Low socioeconomic status is associated with worse survival in children with cancer: A systematic review. PloS One (2014) 9(2):e89482. 24586813 10.1371/journal.pone.0089482 PMC3935876

